# Evaluating the World Health Organization’s health promotion strategies: Sentiments, health beliefs, and the COVID-19 pandemic

**DOI:** 10.1371/journal.pone.0311825

**Published:** 2024-11-18

**Authors:** Di Wang, Zhifei Mao, Xinyu Yao

**Affiliations:** 1 Faculty of Humanities and Art, Macau University of Science and Technology, Macau, China; 2 Disease AI Laboratory on Epidemic and Medical Big Data Instrument Applications, Faculty of Innovation Engineering, Macau University of Science and Technology, Macau SAR, China; 3 School of Media and Communication, Shenzhen University, Shenzhen, China; Yarmouk University, JORDAN

## Abstract

Based on the Health Belief Model (HBM), this study examined what messages about COVID-19 were presented on the World Health Organization (WHO)’s Facebook posts, and evaluated WHO’s health promotion strategies by examining the public engagement and sentiment stimulated by different health promotion constructs. One issue with previous studies on HBM and social networking sites is that many researchers considered positive "online engagements" as evidence of "effective health promotion". However, online engagement measures such as shares and comments cannot reflect the sentiment’s valence. Another limitation in previous studies is that they often failed to differentiate between sentiments towards health measures and sentiments towards the disease. We utilized Facebook’s emojis to explore the public’s distinct sentiments towards the WHO’s COVID-19-related health measures and sentiments towards COVID-19. We used content analysis to examine the all the COVID-19-related Facebook posts published by the WHO in 2020, the first year of the pandemic when the COVID-19 vaccines were not generally available to the public. In general, the use of HBM constructs was successful in capturing users’ attention and generating engagement. However, regarding the effect of the use of HBM constructs on the beliefs in COVID-19-related health measures, the results were complex. The mentioning of the beliefs about a disease (perceived susceptibility and severity) tend to induce people’s negative sentiments. The mentioning of ways to increase self-efficacy and cues to actions significantly reduced people’s online engagements and sentiment (either positive or negative). Benefits only stimulated users’ negative reactions while barriers could not stimulate any reactions. Video and text posts generally attracted more Facebook engagement than image and text posts, while text-only posts generally received the least Facebook engagement. We demonstrated that certain health promotion strategies can backfire and even induce negative reactions. Policymakers should be alert to this phenomenon when mentioning certain constructs during health promotions.

## Introduction

After the COVID-19 outbreak, the World Health Organization (WHO) aimed, in its own words, to prevent the spread of the virus by providing guidance on general and specific topics related to the pandemic [1]. In general, such efforts fell into two categories: (1) define the pandemic in terms of COVID-19 transmission, symptoms, and severity in order to identify persons at higher risk of developing serious illness; and (2) suggest the health measures needed to prevent and cure the virus and to end the pandemic, both non-pharmaceutical (e.g., social distancing, wearing masks) and pharmaceutical (e.g., medical treatments, vaccines; WHO n.d.). The WHO’s Facebook account constantly circulated these two categories of guidance, thereby promoting knowledge and advice on COVID-19 to a global audience.

But were these promotion efforts successful? Studies have shown that since the COVID-19 outbreak, public trust in the WHO and governments all around the world has faced constant challenges [2, 3]. Distrust of experts’ judgment on the severity—or even the existence—of the virus, the social measures to prevent its transmission, and the vaccines used to combat COVID-19 spread on social networking sites[4, 5]. Another challenge to the WHO’s promotion of their advice on COVID-19 is that some people have simply underestimated or ignored the pandemic[6, 7].

As a result, it is important for health experts and policy-makers to know which communication strategies work to promote health-related beliefs and actions on social networking sites. In order to evaluate these actions, this study used the Health Belief Model (HBM) as a framework to examine what kind of posts about COVID-19 on the WHO’s Facebook page triggered what kind of public engagement and sentiment. We further explored the effect of media richness on public engagement and sentiment.

The HBM theory assumes that humans will adopt health-related behaviors when they are aware of the severity and vulnerability of a disease and its relevant health measures [8, 9]. To be more specific, such a belief includes the following perceptions: (1) susceptibility, i.e. whether a person is vulnerable to a disease or health risk, (2) severity, i.e. the severity of a disease, (3) barriers, i.e. the difficulty of health-related actions, (4) benefits, i.e. the benefits of taking those actions, (5) self-efficacy, i.e. whether a person can successfully implement the recommended health behavior, and (6) cues to actions, i.e. stimulus cues that trigger individuals to engage in appropriate health behaviors [8–10].

Among these six factors, perceived susceptibility and severity are related to the beliefs about a disease, and perceived barriers, benefits, self-efficacy, and cues to actions are related to beliefs in related health measures. Such a framework fits with the WHO’s circulation of COVID-19 guidance online, which also emphasized the definition of the pandemic and the promotion of health measures. Based on this theoretical fit, we argue that it is possible to evaluate the WHO’s self-promotion of its COVID-19 guidance systematically using the theoretical framework of the HBM. We can test whether the mention of perceived susceptibility and severity echoed viewers’ beliefs about the disease and whether the mention of perceived barriers, benefits, self-efficacy, and cues to actions received viewers’ support in relation to the health measures. While regarding the above six factors as independent variables, this research’s independent variables are people’s engagements with WHO online, including not only “share”, “comment”, and “like”, but also the detailed sentiments of online networking users. To examine the sentiments of users in a HBM framework makes this study different from previous research of HBM. We will elaboratively explain the reasons to include sentiment analysis in a HBM research in the next section of this paper. Furthermore, we also consider of media richness (e.g., whether to use visuals in an online message while promoting COVID-19 related policies) as an influential independent variable in affecting people’s online enablement in respond to the health promotion online.

The contribution of the current study is threefold. First of all, it explored the public sentiment and engagement in COVID-19-related posts published by the WHO on Facebook for the first time. Therefore, this study fills a gap in the knowledge about public sentiment toward the WHO’s COVID-19 approach. Secondly, previous studies on the public’s sentiment towards COVID-19 policy usually did not distinguish between the sentiments towards health measures and sentiments towards the disease [11]. This study is the first to investigate the public’s sentiments towards these two different aspects. Finally, this study used emoticon data from Facebook for sentiment analysis. Compared with previous studies that used machine learning to analyze sentiments [12], emoticons clicked by users can more accurately reflect users’ sentiments.

### Measuring the effect of the HBM: Sentiment analysis on social networking sites

Surveys have traditionally been used to test people’s opinions and reactions toward the promotion of particular health behaviors [13, 14]. In recent years, aware of the impact of social media on health promotion, scholars have implemented the HBM to evaluate posts and activities related to health measures on popular platforms such as Twitter[11, 15, 16] and Facebook [17, 18]. These studies evaluated the impact of health promotion strategies by examining which constructs of the HBM increased people’s responses toward certain health promotions. The results of these studies varied, but the majority agreed that the HBM constructs worked to predict social media engagements in a case-by-case way. Some constructs of the HBM might be more effective than others in triggering online engagement regarding health measures designed to tackle a certain health problem. This might be due to the individual characteristics of a health problem and the relevant health measures. For example, the effectiveness of the HBM in promoting health measures against a non-communicable disease could differ from its effectiveness when the disease is communicable [19]. In this study, we focused on COVID-19 and its related health measures circulated by the WHO’s Facebook page.

A major problem with previous studies on the HBM and social networking sites is that many scholars regarded positive “online engagements” as evidence of the “effectiveness of health promotion” when implementing the HBM online. However, sentiment analysis on social networking sites has demonstrated that online engagements showed not only people’s support and agreements but also their negative emotions, such as anger, sadness, and disagreement [20, 21]. The ways that people can express their emotions on social media depend on the features of the related platforms. Facebook, for example, provides its users with different emoticons to react to a post (e.g., “like”, “love”, “ha-ha”, “wow”, “sad”, and “angry”). Researchers of sentiment analysis have found that Facebook users used such emoticons to express their positive and negative feelings, and such expressions could predict the overall attitude of the users toward a social issue[22–24]. If a user reacted to a health promotion post with “angry,” for instance, the user’s intention might be interpreted as opposing, not supporting, the health promotion. Thus, examining the effect of using the HBM constructs on Facebook, as in our study, not only sheds light on which constructs contribute to people’s positive reactions to health promotions, but also examines which constructs contribute to negative responses. Such an analysis is especially important regarding the controversial health measures recommended during the COVID-19 pandemic.

Accordingly, in this study, we implemented the HBM constructs to examine health promotion messages related to COVID-19 on Facebook. To further enrich the HBM, we also utilized the insights of sentiment analysis. We raise the following questions:

Research Question (RQ)1: To what extent did the HBM constructs (perceived susceptibility, severity, benefits, barriers, self-efficacy, and cues to action) appear in the WHO’s posts about COVID-19 on Facebook?
RQ2: Did the presence of the HBM constructs (perceived susceptibility, severity, benefits, barriers, self-efficacy, and cues to action) in the WHO’s posts about COVID-19 on Facebook have an impact on users’ sentiments?

### Media richness and social media engagement

Due to its professional nature, health information is often difficult for ordinary people to understand. When trying to convey complex information, it can be helpful to use visuals (Lipkus, 2007). Pictures adjacent to text can enhance an individual’s attention, comprehension, and recall of health information compared to text alone (Houts, Doak, Doak & Loscalzo, 2006). Hamaguchi et al. (2020) also showed that validated graphical data or chart presentations can be an effective tool for providing medical information during a pandemic. Moreover, studies have found that text and graphic health warnings are more effective than plain text warnings (Rosenblatt, Dixon, Wakefield & Bode, 2019). Rus and Cameron (2016) found that Facebook posts about diabetes with images attracted more liking and sharing than posts without images.

In addition to images, video is another mainstream form of multimedia, and its effectiveness in improving the interaction of netizens in health communication has also been proven. Kite et al. (2016) found that video posts were the most engaging type of post when studying the characteristics of highly engaged posts on the Facebook pages of public health organizations in Australia, and text-only posts received fewer likes and shares than images type of posts.

Past studies have shown that posts containing visuals in health communication will attract more Internet users’ participation than otherwise. However, few scholars have compared whether there are differences between specific visual types and viewers’ participation, especially in the field of health communication. Therefore, in this study, we want to explore whether which level of media richness they used text-only or included images or videos) might have had the highest impact on people’s users’ engagement and raise the following questions:

RQ3: What was the media richness level (text-only, image and text, and video and text) of WHO’s Facebook posts about COVID-19?
RQ4: Which level of media richness (text-only, image and text, or video and text) led to the highest Facebook user engagement?

## Method

A content analysis was conducted with COVID-19-related posts from the WHO’s Facebook account from January 1, 2020, to December 31, 2020. We declare that the dataset for this study consisted of publicly available social media posts on Facebook. To ensure ethical use of the data, we adhered strictly to the platform’s terms of service and data usage policies. Our collection and analysis methods complied with the terms and conditions for the source of the data. We did not collect any private user information, and all data used in this study was publicly accessible at the time of collection. To protect user privacy, we anonymized all data by removing personally identifiable information before analysis.

Since COVID-19 infection cases were not reported in many countries until January 2020, the WHO declared the outbreak of the novel coronavirus as a global health emergency on January 30, 2020. Before the availability of COVID-19 vaccines in December 2020, governments around the world developed various policies of non-pharmaceutical interventions (NPIs) to combat the virus [25]. These policies were different from country to country (such as whether to lock down cities), and some policies were also different from what the WHO advocates (such as whether one should wear a mask) [25]. The public is confused about the appropriate measures to combat the virus [26]. Therefore, we focused on the first year of the COVID-19 pandemic, aiming to explore to what extent the WHO’s health promotion works before the general availability of COVID-19 vaccines. Therefore, we selected the whole year of 2020 as our sample.

In March 2021, DiVoMiner^®^ was used to retrieve the WHO’s Facebook posts about COVID-19. Posts that contain “COVID/COVID19/COVID-19/COVID2019/coronavirus/corona virus” were selected. After removing irrelevant posts, a total of 673 posts were included in the analysis. We used the entire population of the 2020 data, so we did not use sampling methods. The Facebook engagement variables and sentiment variables were downloaded from the Facebook website including the number of times “hit”, “comment”, “share”, “like”, “love”, “wow”, “ha-ha”, “sad”, and “angry” appeared.

Each Facebook post was examined for one or more of the HBM constructs, susceptibility, severity, benefits, barriers, cues to action, and ways to increase self-efficacy. Since the HBM was originally proposed to measure psychological variables, we adjusted the operational definition of the HBM constructs according to that of [27] to create variables capable of measuring media content. For the operationalization of the HBM constructs, see Table 1. All six items were coded as 1 when mentioned and 0 when not mentioned. The media form variable was coded as text = 1, image and text = 2 and video and text = 3 (there were no other forms such as picture and video without texts).

**Table 1:** Operational definition of the HBM constructs.

HBM constructs	Operational definition	Example
Susceptibility	Define the population at risk and the risk level. Personalize the risk according to a person’s characteristics or behavior. Increase susceptibility if it is too low.	The evidence we have suggests that those over sixty are at the highest risk.
Severity	Describe the consequences of the risk.	Smokers are more likely to have a severe case of COVID-19.
Benefits	Clarify the positive impact of the recommended measures in reducing the severity of risks or consequences.	#COVID19 can be spread by people who do not have symptoms & do not know that they are infected. Masks worn over the mouth AND nose can help prevent people who have COVID-19 from spreading the virus to others.
Barriers	Determine the physical and psychological costs of the proposed action.	Some people with disabilities might experience barriers to implementation. For example, perhaps it is more difficult for them to access a sink or to rub their hands together.
Cues to action	Remind people to take action.	We need to practice isolation, we closed borders.
Ways to increase self-efficacy	Provide training and guidance on implementation actions.	We say one to two meters, but that doesn’t translate very well, but yes, three feet to six feet away from another individual.

Note: The HBM is short for the Health Belief Model.

Two graduate students who were fluent in English coded all the files. We calculated the inter-coder reliability of the two coders by double-coding a random subsample (n = 146 or 22%) of the data. Krippendorf’s alpha ranged from 0.85 to 0.97 for the six HBM constructs.

Descriptive analysis was used to answer RQ1 and RQ3. As the Facebook engagement variables were not normally distributed, nonparametric Mann-Whitney U tests were used to examine the relationship between the presence of the HBM constructs and Facebook engagement variables, in response to RQ2. As media form includes three forms, accordingly, a series of nonparametric Kruska-Wallis H tests were run to examine which media form led to more Facebook engagement and sentiment, in response to RQ4.

## Results

Of the 670 posts in the sample, 72.4% (n = 485) contained HBM constructs. The most frequently used HBM construct was cues to action (77.8%, n = 382), followed by susceptibility (22.1%, n = 107), severity (19%, n = 92), ways to increase self-efficacy (18.4%, n = 89), benefits (16.1%, n = 78), and barriers (8.0%, n = 39).

### Susceptibility and Facebook engagement and sentiment

As can be seen in Table 2, in general, the mentioning of susceptibility led to more hit, comment, sad and angry, while the mentioning of susceptibility led to fewer share and like.

**Table 2:** Susceptibility and Facebook engagement and sentiment.

Engagement variable	Mean rank with vulnerability present	Mean rank with vulnerability absent	Mann-Whitney U	*P* value	Z
Hit	269.51	215.21	6,979.00	<.001	-3.72
Comment	400.84	323.08	23,129.50	<.001	-3.81
Share	289.08	344.32	25,154.00	.007	-2.71
Like	289.61	344.22	25,210.50	.007	-2.68
Love	338.15	335.00	29,836.50	.88	-0.16
Wow	338.94	334.85	29,752.00	.84	-0.20
Ha-Ha	320.21	338.41	28,484.50	.37	-0.89
Sad	403.16	322.64	22,881.00	<.001	-3.95
Angry	420.57	319.33	21,017.50	<.001	-4.96

Specifically, posts emphasizing susceptibility to COVID-19 (mean rank = 269.51) were hit on more than posts that did not emphasize susceptibility (mean rank = 215.21), U = 6,979.00, *p*<0.001. Posts emphasizing susceptibility to COVID-19 (mean rank = 400.84) were commented on more than posts that did not emphasize susceptibility (mean rank = 323.08), U = 23,129.50, *p*<0.001. Posts emphasizing susceptibility to COVID-19 (mean rank = 403.16) induced more “sad” reactions than posts that did not emphasize susceptibility (mean rank = 322.64), U = 22,881.00, *p*<0.001 and posts emphasizing susceptibility to COVID-19 (mean rank = 420.57) induced more “angry” reactions than posts that did not emphasize susceptibility (mean rank = 319.33), U = 21,017.50, *p*<0.001.

In contrast, posts emphasizing susceptibility to COVID-19 (mean rank = 289.08) were shared fewer than posts that did not emphasize susceptibility (mean rank = 344.32), U = 25,154.00, *p* = .007, while posts emphasizing susceptibility to COVID-19 (mean rank = 289.61) were liked fewer than posts that did not emphasize susceptibility (mean rank = 344.22), U = 25210.50, *p*<0.01.

### Severity and Facebook engagement and sentiment

Similar to vulnerability, the mentioning of severity also led to more hit, comment, sad and angry, while the mentioning of severity also led to fewer share and like (Table 3).

**Table 3:** Severity and Facebook engagement and sentiment.

Engagement variable	Mean rank with severity present	Mean rank with severity absent	Mann-Whitney U	*P* value	Z
Hit	263.17	217.70	5,194.50	0.009	-2.62
Comment	407.82	323.99	19,934.50	<0.001	-3.86
Share	274.13	345.27	20,942.00	0.001	-3.27
Like	277.58	344.72	21,259.00	0.002	-3.09
Love	336.24	335.38	26,519.50	0.97	-0.04
Wow	344.34	334.09	25,774.50	0.64	-0.47
Ha-Ha	314.04	338.92	24,613.50	0.25	-1.15
Sad	400.92	325.09	20,569.00	<0.001	-3.49
Angry	428.17	320.75	18,062.50	<0.001	-4.94

Specifically, posts emphasizing the severity of COVID-19 (mean rank = 263.17) were hit on more than posts that did not emphasize severity (mean rank = 217.70), U = 5,194.50, *p* = 0.009. Posts emphasizing the severity of COVID-19 (mean rank = 407.82) were commented on more than posts that did not emphasize severity (mean rank = 323.99), U = 19,934.50, *p*< 0.001. Posts emphasizing the severity of COVID-19 (mean rank = 400.92) also induced more “sad” reactions than posts that did not emphasize severity (mean rank = 325.09), U = 20,569.00, *p*<0.001. In addition, posts emphasizing the severity of COVID-19 (mean rank = 428.17) induced more “angry” reactions than posts that did not emphasize severity (mean rank = 320.75), U = 18,062.50, *p*<0.001.

Posts emphasizing the severity of COVID-19 (mean rank = 274.13) were also shared fewer than posts that did not emphasize severity (mean rank = 345.27), U = 20,942.00, *p*<0.01 and posts emphasizing the severity of COVID-19 (mean rank = 277.58) were liked fewer than posts that did not emphasize severity (mean rank = 344.72), U = 21,259.00, *p* = .002.

### Benefit and Facebook engagement and sentiment

Table 4 shows that posts emphasizing the benefits of taking preventative measures induced more hit, comment and “angry” reactions. Specifically, posts emphasizing the benefits of taking preventative measures (mean rank = 303.88) were hit more than posts that did not emphasize the benefit (mean rank = 214.95), U = 3,678.50, *p* <.001. Posts emphasizing the benefits of taking preventative measures (mean rank = 408.44) were commented more than posts that did not emphasize benefit (mean rank = 325.89), U = 17,398.50, *p*<0.001. Posts emphasizing the benefits of taking preventative measures (mean rank = 417.49) also induced more “angry” reactions than posts that did not emphasize benefit (mean rank = 324.70), U = 16,692.50, *p*<0.001.

**Table 4:** Benefits and Facebook engagement and sentiment.

Engagement variable	Mean rank with benefit present	Mean rank with benefit absent	Mann-Whitney U	*P* value	Z
Hit	303.88	214.95	3,678.50	<.001	-4.98
Comment	408.44	325.89	17,398.50	<.001	-3.54
Share	313.65	338.38	21,383.50	.29	-1.06
Like	330.24	336.19	22,678.00	.80	-0.26
Love	369.04	331.08	20,472.00	.10	-1.63
Wow	347.58	333.91	22,146.00	.56	-0.59
Ha-Ha	354.49	333.00	21,607.00	.36	-0.92
Sad	360.94	332.15	21,103.50	.22	-1.24
Angry	417.49	324.70	16,692.50	<.001	-3.98

### Barriers and Facebook engagement and sentiment

Table 5 shows that barriers were not significantly related to any of the Facebook engagement variables.

**Table 5:** Barriers and Facebook engagement and sentiment.

Engagement variable	Mean rank with barriers present	Mean rank with barriers absent	Mann-Whitney U	*P* value	Z
Hit	242.21	220.15	3,243.50	.35	-0.94
Comment	373.78	333.13	10,811.50	.20	-1.27
Share	277.04	339.11	10,024.50	.05	-1.94
Like	284.62	338.65	10,320.00	.09	-1.69
Love	321.42	336.37	11,755.50	.64	-0.47
Wow	323.82	336.22	11,849.00	.70	-0.39
Ha-ha	310.50	337.05	11,329.50	.41	-0.83
Sad	374.86	333.07	10,769.50	.19	-1.31
Angry	376.36	332.97	10,711.00	.17	-1.36

### Cues to action and Facebook engagement and sentiment

Table 6 shows that posts emphasizing cues to action induced more hit but less “wow” and “sad” sentiment.

**Table 6:** Cues to action and Facebook engagement and sentiment.

Engagement variable	Mean rank with cues to action present	Mean rank with cues to action absent	Mann-Whitney U	*P* value	Z
Hit	229.87	211.25	22,206.50	.04	-2.07
Comment	342.49	326.23	52,339.00	.28	-1.08
Share	334.30	337.09	54,549.50	.85	-0.19
Like	323.12	351.91	50,280.50	.06	-1.91
Love	339.23	330.56	53,584.50	.57	-0.57
Wow	321.74	353.75	49,752.50	.03	-2.12
Ha-ha	327.45	346.18	51,932.50	.22	-1.24
Sad	320.09	355.94	49,120.00	.02	-2.37
Angry	341.84	327.09	52,586.50	.33	-0.98

Specifically, posts emphasizing cues to action (mean rank = 229.87) induced more hit than posts that did not emphasize cues to action (mean rank = 211.25), U = 22,206.50, *p* = .04.

By contrast, posts emphasizing cues to action (mean rank = 321.74) induced fewer “wow” reactions than posts that did not emphasize cues to action (mean rank = 353.75), U = 49,752.50, *p* = .034 and that posts emphasizing cues to action (mean rank = 320.09) induced fewer “sad” reactions than posts that did not emphasize cues to action (mean rank = 355.94), U = 49,120.00, *p* = .018.

### Ways to increase self-efficacy and Facebook engagement and sentiment

Table 7 shows that posts emphasizing ways to increase self-efficacy induced significantly fewer Facebook engagement and sentiment in general.

**Table 7:** Ways to increase self-efficacy and Facebook engagement and sentiment.

Engagement variables	Mean rank with ways to increase self-efficacy present	Mean rank with ways to increase self-efficacy absent	Mann-Whitney U	*P* value	Z
Hits	235.55	219.01	9511.00	.23	-1.20
Comment	291.47	342.24	21,936.00	.02	-2.30
Share	331.82	336.06	25,527.00	.85	-0.19
Like	263.78	346.49	19,471.00	<.001	-3.75
Love	283.43	343.48	21,220.00	.006	-2.73
Wow	264.50	346.38	19,535.50	<.001	-3.72
Ha-ha	275.59	344.68	20,522.50	.002	-3.14
Sad	245.67	349.26	17,860.00	<.001	-4.70
Angry	281.58	343.76	21,055.50	.005	-2.82

Specifically, posts emphasizing ways to increase self-efficacy (mean rank = 291.47) were commented fewer than posts that did not emphasize ways to increase self-efficacy (mean rank = 342.24), U = 21,936.00, *p* = .02. Likewise, posts emphasizing ways to increase self-efficacy (mean rank = 263.78) were liked fewer than posts that did not emphasize ways to increase self-efficacy (mean rank = 346.49), U = 19,471.00, *p*<.0.001.

It was also found that posts emphasizing self-efficacy (mean rank = 283.43) induced fewer “love” reactions than posts that did not emphasize ways to increase self-efficacy (mean rank = 343.48), U = 21,220.00, *p* = .006 and that posts emphasizing ways to increase self-efficacy (mean rank = 264.50) induced fewer “wow” reactions than posts that did not emphasize ways to increase self-efficacy (mean rank = 346.38), U = 19,535.50, *p*<.0.001.

In addition, posts emphasizing ways to increase self-efficacy (mean rank = 275.59) induced fewer “ha-ha” reactions than posts that did not emphasize ways to increase self-efficacy (mean rank = 344.68), U = 20522.50, *p* = .002. Posts emphasizing ways to increase ways to increase self-efficacy (mean rank = 245.67) induced fewer “sad” reactions than posts that did not emphasize ways to increase self-efficacy (mean rank = 349.26), U = 17860.00, *p*<.0.001. Finally, posts emphasizing ways to increase self-efficacy (mean rank = 281.58) induced fewer “angry” reactions than posts that did not emphasize ways to increase self-efficacy (mean rank = 343.76), U = 21055.50, *p* = .005.

### Media richness and Facebook engagement and sentiment

About half of the posts consisted of a mix of image and text (41.3%; n = 277), 57.2% (n = 383) consisted of a mix of video and text, and 1.5% (n = 10) consisted of text-only. A series of Kruska-Wallis H tests were run to examine which media form led to more Facebook engagement and sentiment (Table 8). Results showed that there were significant differences between the three forms in attracting hits (H(2) = 236.99, *p*<.001), comments (H(2) = 92.53, *p*<.001), shares (H(2) = 15.50, *p*<.001), “love” (H(2) = 60.51, p<.001), “wow” (H(2) = 16.82, *p*<.001), “ha-ha”(H(2) = 28.45, *p*<.001), “sad” (H(2) = 28.84, p<.001) and “angry” (H(2) = 137.49, *p*<.001). However, there were no significant differences between the three forms in attracting the number of likes, H(2) = 4.61, *p* = .052.

**Table 8:** Media richness and Facebook engagement and sentiment.

Engagement variable	Media Richness	Mean rank of engagement variable	Kruska-Wallis H	*P* value	df
Hit	Text-only	169.50			
	Image and text	170.28			
	Video and text	315.57	236.99	<.001	2
Comment	Text-only	218.25			
	Image and text	253.79			
	Video and text	397.66	92.53	<.001	2
Share	Text-only	98.10			
	Image and text	343.35			
	Video and text	336.02	15.50	<.001	2
Like	Text-only	233.40			
	Image and text	325.19			
	Video and text	345.62	4.61	.052	2
Love	Text-only	144.95			
	Image and text	275.32			
	Video and text	384.00	60.51	<.001	2
Wow	Text-only	247.80			
	Image and text	302.82			
	Video and text	361.43	16.82	<.001	2
Ha-ha	Text-only	264.45			
	Image and text	290.47			
	Video and text	369.92	28.45	<.001	2
Sad	Text-only	272.60			
	Image and text	289.77			
	Video and text	370.22	28.84	<.001	2
Angry	Text-only	281.10			
	Image and text	232.75			
	Video and text	411.23	137.49	<.001	2

We also ran a series of pairwise comparisons using Dunn’s test to examine which form led to the most engagement. Results showed that video and text posts generally are the most effective form in attracting engagement. Specifically, in terms of hits, video and text posts (Mean rank = 315.57) attracted more hits than text-only posts (Mean rank = 169.50), p <.001 and that image and text posts (Mean rank = 170.28) also attracted more hits than text-only posts (Mean rank = 169.50), *p*<.001.

In terms of comments, video and text posts (mean rank = 397.66) attracted more comments than text-only posts (mean rank = 218.25), *p* = .011 and that video and text posts (mean rank = 397.66) also attracted more comments than image and text posts (mean rank = 253.79), *p*<.001.

In terms of shares, video and text posts (Mean rank = 336.02) attracted more shares than text-only posts (mean rank = 98.10), *p*<.001; video and text posts (mean rank = 343.35) attracted more shares than image and text posts (Mean rank = 98.10), *p*<.001.

In terms of “love”, video and text posts (Mean rank = 384.00) attracted more “love” than text-only posts (Mean rank = 144.95), *p*<.001 and that video and text posts (Mean rank = 384.00) also attracted more “love” than image and text posts (Mean rank = 275.32), *p*<.001.

Finally, video and text posts (mean rank = 361.43) attracted more “wow” than image and text posts (mean rank = 302.82), *p*<.001. Video and text posts (mean rank = 369.92) attracted more “ha-ha” than image and text posts (mean rank = 290.47), p<.001. Video and text posts (Mean rank = 370.22) attracted more “sad” than image and text posts (Mean rank = 289.77), *p*<.001. Video and text posts (Mean rank = 370.22) attracted more “angry” reactions than image and text posts (Mean rank = 289.77), *p* <.001.

## Discussion

The HBM constructs of perceived susceptibility and perceived severity, which both relate to beliefs in COVID-19, stimulated similar online engagements and sentiments (e.g., shares, comments, likes, sadness, and anger) by Facebook users.

People were willing to read and comment on posts that mentioned the severity and susceptibility of COVID-19, and expressed sad and angry reactions to these posts. However, they were less likely to share and like these posts. In other words, when people are told about their high susceptibility to COVID-19 and that the severity of COVID-19 is high, they tend to show anger and sadness. These reactions in our study not only show people’s negative sentiments towards the disease, but also demonstrate the effectiveness of the HBM constructs in influencing people’s beliefs about COVID-19 and educating them about what the pandemic is and its consequences. Overall, we found that the HBM constructs of perceived susceptibility and perceived severity boosted the WHO’s health promotion related to beliefs about COVID-19: from what it is to what it causes.

However, regarding the constructs related to health measures (i.e. perceived benefits, ways to increase self-efficacy, cues to actions, and perceived barriers), the results were mixed. The construct of perceived benefits did not trigger any notable positive responses, it stimulated the reaction of “anger.” It is worth noting that in response to the posts that talked about the severity and vulnerability of the disease, “anger” could indicate people’s upset about the consequences that the disease caused, and not necessarily their denial of the severity and vulnerability of the disease. However, when people reacted to posts about health measures or policies with “anger,” it reflected a strong negative attitude against those measures. Previous studies have demonstrated that people use “anger” to express their rejection of a policy or fury toward a policymaker[21, 28]. In the context of the pandemic, we argue that people’s “angry” reactions mean that the construct of “perceived benefits” stimulated people’s fierce opposition to the health measures against COVID-19 mentioned in the posts.

Compared with single negative response toward perceived benefits, the mentioning of ways to increase self-efficacy, and cues to actions generally reduced people’s online engagements and sentiment (either positive or negative) comparing to other posts. The use of these two constructs seems to backfire, as readers may be viewed them as manipulation and may decrease people’s reactions. A similar finding was reported by Ashwell and Murray [29], which found that while the news report about vaccination were generally positive in Australia and New Zealand between 2016 and 2017, the vaccination rate in New Zealand was decreasing during this period. They argued that positive reports about vaccination might be viewed as advertising and the public may resist such manipulation.

Unlike the other five constructs, barriers did not elicit many reactions from Facebook users. Compared to other posts, people seemed indifferent to those mentioning barriers. The obstacles to taking preventive measures vary by country. Some nations may lack basic medical facilities and materials such as ventilators and masks, while others may face shortages of doctors and nurses. Consequently, the barriers mentioned by the WHO may not align with the needs of specific target countries. As a result, these posts did not receive many responses.

The data on media richness indicated that inserting a video into the WHO’s posts increased all online engagement and sentiment on Facebook, except for “like”. It is noteworthy that the impact of video was also a mixture of negative and positive. While it stimulated positive responses, such as “like” and “wow”, it also triggered “anger” and “sad” reactions. This finding is echoed by previous studies of media richness, which determined that the use of images and videos can stimulate more reactions from social media users [30].

Some limitations need to be pointed out before we make our conclusion. One major limitation is the ambiguity of Facebook’s response features. The interpretation of certain reactions, such as the "haha" emoji, may vary greatly depending on the context and may indicate true happiness or ridicule. This ambiguity emphasizes the necessity for a more detailed study of the meaning of each reaction in different context. Future research should systematically explore the attitudes behind these reactions, possibly through qualitative methods such as interviews or focus groups, to gain a deeper understanding of users’ intentions and emotions. The study’s exclusive focus on Facebook also limits the generalizability of the findings to other social media platforms. To address this issue, future research can conduct comparative studies on multiple platforms such as Twitter, Instagram, and TikTok. These studies can reveal platform specific impacts and provide insights into how the effects of the same health communication strategy vary in different social media environments.

## Conclusion

We conclude that in the context of the World Health Organization’s (WHO) communication about COVID-19 on Facebook, the Health Belief Model (HBM) constructs were effective in shaping users’ beliefs about the pandemic. People were inclined to disseminate the WHO’s information about COVID-19, such as the details about the illness and its potential severity. However, regarding the effect of the use of HBM constructs on the beliefs in COVID-19-related health measures, the results were mixed and more complex.

On one hand, while some HBM constructs promoting health measures sparked public participation online, Facebook users’ reactions to these posts were not uniformly positive. Notably, the constructs of ways to increase self-efficacy and cues to action reduced both positive and negative reactions. The construct of perceived benefits, in particular, only stimulated users’ negative reactions, which was an unexpected and concerning outcome.

Our study also revealed that the WHO’s use of visual media, such as images or videos, in health promotion posts significantly stimulated all indicators of users’ online engagement. These multimedia elements elicited both positive and negative sentiments, highlighting their power to provoke emotional responses across the spectrum.

In general, the use of HBM constructs was successful in capturing users’ attention and generating engagement. However, our findings underscore a critical insight: certain health promotion strategies can backfire and even induce negative reactions from users. This unexpected outcome emphasizes the complexity of public health communication in social media contexts. Given these nuanced results, policymakers and health communicators should be vigilant when employing specific constructs during health promotions. It is crucial to carefully consider the potential implications of each communication strategy and continuously monitor public responses. Future health communication efforts may benefit from a more tailored approach that takes into consideration the varying impacts of different HBM constructs and message formats on public sentiment and engagement.
